# Post-translational regulation of COX2 activity by FYN in prostate cancer cells

**DOI:** 10.18632/oncotarget.1983

**Published:** 2014-05-18

**Authors:** Anna Alexanian, Bradley Miller, Marla Chesnik, Shama Mirza, Andrey Sorokin

**Affiliations:** ^1^ Division of Nephrology and Kidney Disease Center, Department of Medicine, Medical College of Wisconsin, Milwaukee, Wisconsi, USA; ^2^ Department of Biochemistry, Biotechnology and Bioengineering Center, Medical College of Wisconsin, Milwaukee, Wisconsin, USA

**Keywords:** COX2 regulation/DU145 cells/prostaglandins/phosphorylation/prostate cancer/Src family kinases

## Abstract

While increased COX2 expression and prostaglandin levels are elevated in human cancers, the mechanisms of COX2 regulation at the post-translational level are unknown. Initial observation that COX2 forms adduct with non-receptor tyrosine kinase FYN, prompted us to study FYN-mediated post-translational regulation of COX2. We found that FYN increased COX2 activity in prostate cancer cells DU145, independent of changes in COX2 or COX1 protein expression levels. We report that FYN phosphorylates human COX2 on Tyr 446, and while corresponding phospho-mimetic COX2 mutation promotes COX2 activity, the phosphorylation blocking mutation prevents FYN-mediated increase in COX2 activity.

## INTRODUCTION

Cyclooxygenase (COX) is the rate limiting enzyme in the conversion of arachidonic acid (AA) to prostaglandins (PGs). COX catalyzes the oxidation of AA into PGG_2_ and further reduction into PGH_2_. PGH_2_ is then converted by specific isomerases to the biologically active end products, PGD_2_, PGE_2_, PGF_2_, PGI_2_ and other related eicosanoids [1, 2]. There are two isoforms of COX: constitutively expressed cyclooxygenase 1 (COX1) and cyclooxygenase 2 (COX2), an inducible isoform of the enzyme that predominates in pathologies associated with inflammation, such as cancer [3-9]. PGs produced by COX1 are thought to mediate housekeeping functions, while COX2 expression is selectively expressed in some tissues by growth factors, oncogenes and cytokines, and PGs produced by this isoform contribute to cellular processes such as angiogenesis, invasion and anti-apoptosis. There are multiple studies showing a strong correlation between increased COX2 expression and carcinogenesis both in murine cancer models [10-13], as well as in human solid tumors and premalignant lesions of colon, lung, gastric, breast, squamous cell carcinoma of head and neck, esophageal and prostate[3-9]. Mitogen activated pathways that induce COX2 early gene expression in cancer, involve Ras and MAPK cascades [14-18]. The promoter region of COX2 gene contains binding sites for numerous regulatory transcription factors and activation of various mitogen activated pathways have shown to regulate the expression of this gene at the transcriptional level [16, 19]. Presence of multiple repeated sequences on the COX2 mRNA further regulates the expression of this gene at the post-transcriptional level, through the changes in COX2 mRNA stability[20]. Additionally, it seems that the kinetics of prostaglandin synthesis in mammalian cells does not always correlate with the level of cyclooxygenases expression [21, 22], suggesting that not only the expression of the gene but also the function of this enzyme at the post-translational level is highly regulated. Primary sequence of COX cDNAs suggests several sites for post-translational modification. The enzyme contains multiple glycolysation sites [23, 24] and motifs for phosphorylation by different kinases involved in mitogenic pathways [25, 26]. While less well understood than regulation at the level of transcription, the post-translational regulation of the product of COX2 gene which could occur through protein phosphorylation has also been suggested [22]. It has been shown that chemical modification of tyrosine residues in the active site of the enzyme by nitration abolishes its catalytic activity [28]. Furthermore, a study in cerebral endothelial cells showed that protein tyrosine phosphatase inhibitors rapidly stimulate COX activity, whereas protein tyrosine kinase inhibitors had opposite effect [22]. Nevertheless, no specific kinase has been identified as a kinase capable of COX2 phosphorylation and a recent report addressing direct phosphorylation of COX2 by the serine/threonine protein kinase C (PKC) conclued that COX2 is an unfavorable substrate for PKC [29]

Given the established oncogenic role of COX2 in various forms of human cancers, selective COX2 inhibitors (COXIBs) have been widely utilized, however their use has been recently limited by severe cardiovascular side effects [5, 30-32]. Therefore, identification of novel modulators of COX2 enzyme could open up new therapeutic approaches in targeting this enzyme in human cancers. Here we report a novel post-translational regulatory mechanism of COX2 enzyme activity by FYN.

## RESULTS

### COX2 adducts reveal proteins in close proximity of COX2 in cells

The spontaneous decomposition of prostaglandin H_2_, an immediate product of COX2 activity, results in production of γ-keto aldehydes – levuglandins, which are capable of covalently cross-linking different proteins together through their Lys residues [33, 34]. The formation of covalently-linked adducts as detected by western blot analysis with anti-COX2 antibodies was observed both in human cells expressing COX2 as a result of adnovirus-mediated gene transfer and in cells expressing endogenous COX2 as a consequence of cell exposure to mitogenic stimulus. To identify the proteins in molecular complex with COX2, we have purified COX2 cross-linking adducts by affinity chromatography methods and identified their nature using mass spectrometry.

**Figure 1:**
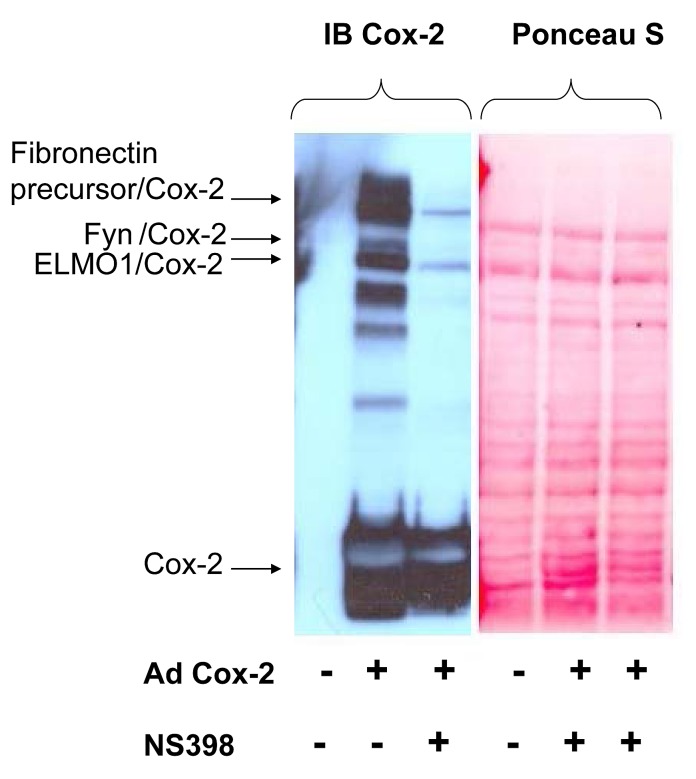
Identification of covalent adducts between COX2 and unknown proteins HMC cells were either left uninfected (-) or infected with AdCOX2 (+) and were incubated either in the absence (-) or presence (+) of COX2 inhibitor NS398. 48h after cells were lysed, subjected to SDS-PAGE, prior to transfer to PVDF membrane and immunoblotted with COX2 antibodies. Equal loading was confirmed by Ponceau S staining as indicated on the left. The nature of COX2 adducts was further uncovered by LC-MS-MS analysis after COX2 was purified by affinity chromatography. Positions of the COX2 band, representing SDS-PAGE resolved not cross-linked monomer COX2, as well as COX2 in complex with FYN, Fibronectin precursor and ELMO1 are shown.

A search against the mammalian database identified a number of proteins found in the molecular complex with COX2 in human mesangial cells (HMC). These proteins included Fibronectin precursor, Engulfment and cell motility protein 1 (ELMO1) and a member of Src family cytoplasmic tyrosine kinases FYN (Fig.1). Mass spectrometry analysis of adducts confirmed the presence of COX2 in high molecular mass bands recognized by anti-COX2 antibodies. It is important to note that the formation of COX2 adducts is observed only in the absence of NS398 treatment, indicating that cyclooxygenase activity is required for the formation of covalent adducts. We have previously shown that ELMO1-mediated COX2 activity enhanced fibronectin expression in HMC cells [35]. Analysis of COX2/Fibronectin precursor interaction did not reveal any regulatory significance (data not shown). Here we have investigated the possibility that FYN, found in complex with COX2, is capable to regulate the enzyme activity. Since it has been reported that COX2 and FYN both are localized in caveolae like structures in some cancer cells [36, 37], it is possible that caveolae is one of cellular comparmtent where FYN is capable to regulate COX2 activity.

### FYN increases activity of the COX enzyme in DU145 cells without changes in COX steady-state protein levels

**Figure 2:**
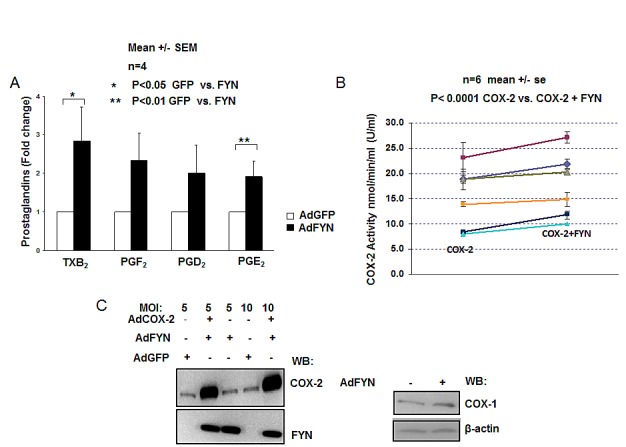
FYN increases COX2 activity independent of changes in COX gene expression (A) FYN increases cyclooxygenase activity resulting in elevated production of prostaglandins in DU145 cells. DU145 cells infected with either adenovirus encoding GFP (white bars) or FYN (black bars) were homogenized, incubated with 40 μM AA, in the presence of 100% O_2_ for 1h, and levels of prostaglandins measured by LC- ESI-MS as described in experimental procedures. Fold change in PG levels from FYN overexpressing cells relative to GFP infected cells is shown. * indicates statistically significant difference in prostaglandin levels in FYN vs. GFP infected cells (n=4). (B) FYN increases activity of COX2 enzyme *in vitro*. *In vitro* kinase assays were performed using recombinant COX2 and recombinant active FYN kinase, followed by measurement of COX2 enzymatic activity by endpoint peroxidase assay as described earlier in experimental procedures. The units of COX2 enzymatic activity is reported as nmol/min/ml (U/ml). Six independent experiments are shown (n=6) each experiment containing triplicate samples. * shows statistically significant difference when comparing COX2 vs. COX2+FYN group. (C) FYN overexpression does not increase endogenous expression of COX genes in DU145 cells. DU145 cells transduced with the indicated adenoviruses (moi of 5 or 10) were lysed and expression of COX1 or COX2 was detected by immunoblotting. β-Actin was used as a loading control, and FYN overexpression was confirmed by immunoblotting with FYN antibodies.

Since COX2 has been shown to have tumorogenic effects in human prostate cancer epithelial cells [38-43], we have analyzed FYN-mediated effect on COX mediated production of prostaglandins in DU145 prostate cancer cells treated with 40 μM AA. DU145 cells with increased FYN signaling generated twice much amount of a variety of prostanoids when compared to GFP expressing cells (Fig. 2A). To compensate for breakdown of lipid byproducts independent of enzymatic activity, the prostaglandin levels were similarly also measured in intact cells, further confirming the increase of FYN effect on COX activity in the prostate cancer cells (data not shown). Additionally, the increase in prostaglandin levels was reduced with selective COX2 inhibitor NS398 (data not shown), and an *in vitro* COX2 endpoint peroxidase assay showed increased COX2 activity in the presence of FYN, indicating that effect of FYN is partially mediated through COX2 isoform of the enzyme (Fig. 2B).

Further, immunoblotting with COX2 and COX1 specific antibodies, showed similar endogenous COX2 and COX1 steady-state protein levels in DU145 cells in the presence or absence of FYN signaling (Fig. 2C), suggesting that FYN regulation of COX2 activity does not occur at the level of regulation of protein expression.

### COX2 is a substrate for direct phosphorylation by oncogenic Src family kinases

**Figure 3:**
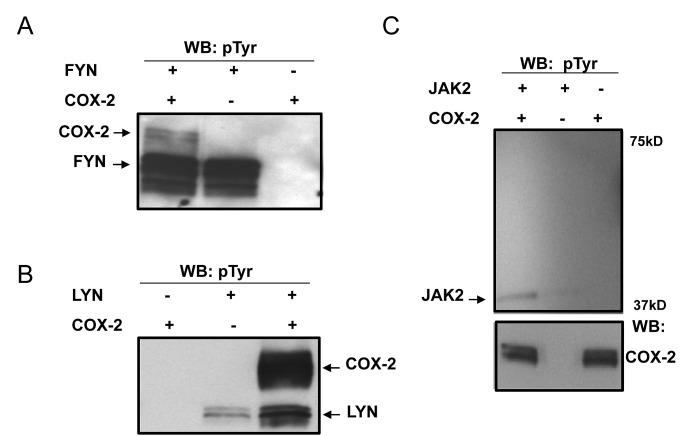
COX2 is a substrate for direct phosphorylation by Src family kinases *in vitro*. *In vitro* kinase assays were carried out using recombinant COX2 as a substrate for recombinant active (A) FYN, (B) LYN or (C) JAK2 kinases as described previously in experimental procedures, followed by detection with non-selective phosphor-tyr specific antibodies. Negative controls included no kinase and no substrate reactions. COX2 phosphorylation, as well as kinase autophosphorylation is shown. Each panel is a representative blot of three experiments and immunoblotting with COX2 antibodies is shown as an equal loading control.

As we have not observed any changes in the expression of the COX isoforms in the presence of FYN, we next checked whether COX2 could be a subject to post-translational modification by FYN. *In vitro* kinase assays were carried out with recombinant COX2 and recombinant active FYN kinase in the presence of ATP for 1h at room temperature, and immunoblotting with non-selective phosphor-tyr specific antibodies was used to detect COX2 phosphorylation. We showed that FYN as well as another Src family member LYN phosphorylate COX2 under these conditions, while an unrelated kinase JAK2 was not able to phosphorylate the enzyme (Fig. 3 A, B, C). Radiolabelled *in vitro* kinase assays carried out in a similar manner in the presence of γ- ^32^P ATP (PerkinElmer), further confirmed COX2 phosphorylation by FYN and LYN kinases (data not shown).

### FYN and LYN kinases phoshorylate COX2 on two distinct tyrosine residues

**Figure 4:**
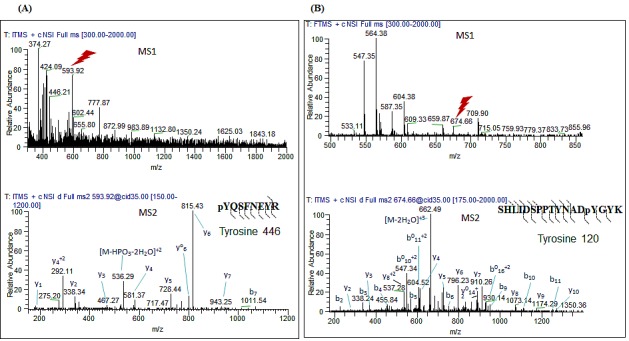
FYN and LYN kinases phosphorylate COX2 on two distinct residues *in vitro*. *In vitro* kinase assays were performed using recombinant COX2 as a substrate for recombinant active (A) FYN or (B) LYN kinases as described previously in experimental conditions, followed by multi-stage fragmentation mass spectrometry as described previously in experimental conditions, to detect exact phosphorylation sites. Representative MS^2^ spectra of the COX2 phosphopeptides pYQSFNEYR and SHLIDSPPTYNADpYGYK.

**Figure 5:**
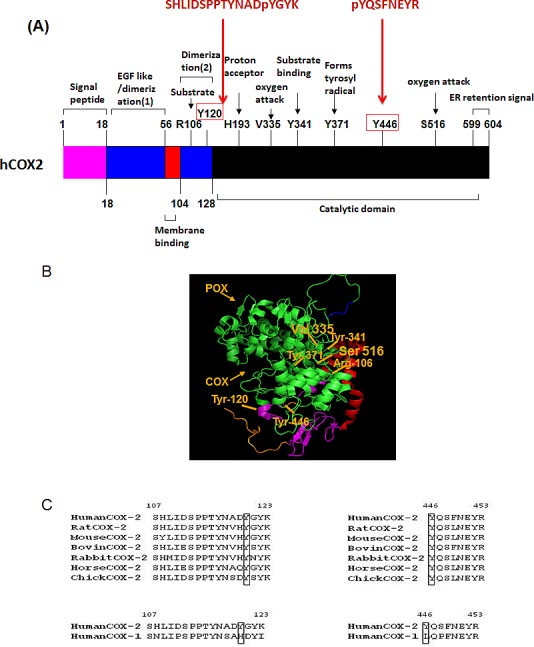
FYN and LYN phosphorylation sites on COX2, located on catalytic and dimerization domains respectively are evolutionary conserved in COX2 from different species, but are absent in the COX1 isoform (A) General linear structure of human COX2 protein functional domains and catalytic residues. COX2 has four domains: amino terminal signal peptide, dimerization domain/EGF like domain, membrane binding domain and catalytic domain. Important amino acid residues required for enzyme catalysis or substrate binding are shown. The catalytic domain contains V335 and S516 which is essential for governing the stereochemistry of oxygen attack at carbon 15 in the production of PGG_2_, Y371, which forms a tyrosyl radical, abstracts hydrogen from the pro-S side of carbon 13 of AA, H193 which is the proton acceptor, and Y341 which is essential for AA binding. R106 located on the dimerization domain of the enzyme is also essential for substrate binding. Indicated are also the sequences of the peptides and positions of the tyrosine phosphorylation sites Y446 and Y120 identified by mass spectrometry. B) The theoretical crystallographic model of human COX2 structure was taken from Protein Data Bank file 1V0X. Y446 and residues involved in the catalysis of COX2 were projected on the crystal structure of COX2 using pYMOL. Functional domains are shown as well: 1) Signal peptide (orange); 2) Dimerization domain (purple); 3) Membrane binding domain (red); Catalytic domains (COX and POX) (green); Membrane targeting sequence (blue). (C) Multiple sequence analysis of tyrosine phosphorylation residues on COX2 enzyme. Sequences of known different species of COX2 and COX isoforms, aligned around the sequences surrounding Y446 and Y120 residues using Uniprot.

To further characterize the exact sites of COX2 phosphorylation by these Src family members, *in vitro* kinase assays were performed as described above, followed by multi-stage fragmentation MS analysis. We identified two distinct phosphorylation sites on the COX2 enzyme, Y446 and Y120, phosphorylated by FYN and LYN kinases respectively (Fig. 4A and 4B). Projection of these phosphorylation sites on the crystal structure of human COX2, showed that Y446 and Y120 are located on the catalytic and dimerization domains of the enzyme respectively (Fig. 5A and 5B). Additionally, multiple sequence alignment analysis revealed that both of these residues are evolutionary conserved in the COX2 enzyme, and are only present in the inducible isoform of COX2 but not the constitutive COX1 isoform of the enzyme (Fig. 5C). The position of these tyrosine sites in the crystal structure of the COX2 enzyme, as well as the fact that it is conserved in the different species, led us to further investigate the importance of these residues in regulation of COX2 activity.

### FYN phosphorylation of Y446 residue in the COX2 enzyme leads to increased activity of the enzyme

**Figure 6:**
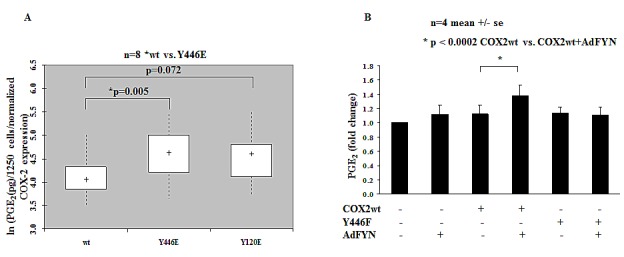
Phosphorylation of Tyrosine Y446 residue on COX2 leads to increased activity of the enzyme (A) Y446E mutant shows increased enzymatic activity when compared to wt COX2 in HEK 293T cells. HEK 293T cells expressing wt or indicated COX2 phosphorylation mutants were stimulated with 30 μM AA for 15 min in serum-free DMEM media and PGE_2_ levels were measured by PGE_2_ Elisa Kit as described earlier in experimental procedures. PGE_2_ levels are shown as pg/1250 cells on a logarithmic scale after normalization to COX2 expression for the different mutants. * represents COX2 mutants demonstrating statistically significant difference in PGE_2_ levels when compared to wt COX2 (n=8). (B) Fyn increases activity of wt COX2 but not Y446F mutant in DU145 cells. PGE_2_ levels were measured in DU145 cells expressing wt or COX2 mutants in the presence or absence of FYN as described above. Fold change in PGE_2_ levels is shown (n=4). * indicates statistically significant difference when FYN+COX2 wt is compared to Null+COX2 wt group.

To address the significance of the identified phosphorylation sites, we used site directed mutagenesis to generate either glutamate (E) or phenalanine (F) mutants of these sites to mimic or block phosphorylation respectively and assessed their affect on COX2 activity. We have found that when Y446 residue was mutated to glutamic acid (E) to mimic negative charge associated with tyrosine phosphorylation, Y446E mutant resulted in increased activity of COX2 compared to wild type (WT) COX2 when overexpressed in HEK 293T cells (Fig. 6A). Furthermore, FYN increased activity of WT COX2 but not of Y446F, the non-phosphorylatable phenalanine (F) mutant of Y446, when expressed in DU145 cells (Fig. 6B). Our data suggest that phosphorylation of COX2 on Y446 residue, leads to increased activity of the enzyme. However, it is still not clear whether effect of phosphorylation at the Y446 site on the activity of the enzyme is direct, allosteric, or rather controls subcellular localization of COX2 and its association with other proteins.

## DISCUSSION

Increased COX2 expression and function has been linked to the initiation and progression of many human cancers, including prostate cancer [3-9, 44-46]. However, described mechanisms involved in the regulation of COX2 expression in cancers are mainly dependent upon transcription [14, 15, 17, 18, 20, 47-52]. The major finding of this paper is that COX2 activity can be regulated also at the post-translational level in prostate cancer, through direct phosphorylation by the oncogenic kinase of the Src family FYN. This conclusion is based on the following findings: 1) FYN is found in close proximity to COX2; 2) FYN increases COX mediated production of prostaglandins independently of altering COX1 or COX2 steady state protein levels; 3) FYN effect on prostaglandin levels is at least partially mediated through COX2 isoform; 4) COX2 is a substrate for direct phosphorylation by FYN and FYN phosphorylation of COX2 leads to increased activity of the enzyme. Even though the formation of COX2 adducts is the consequence of COX2 overexpression, it provides insight into the nature of proteins which are located in close proximity of COX2. We have also observed formation of COX2 adducts with endogenous COX2, when it is overexpressed as a result of activation of mitogen induced pathways (data not shown).

Here we show that FYN is found in close proximity to COX2 to directly regulate its activity. Since COX2 and FYN have been shown to be localized in caveolae like structures in some cancer cells [36, 37], we speculate that caveolae is one possible site where FYN could be found in the same cellular environment as COX2. However, if this is the case, it is still unclear how COX2 leaves its ER residence and migrates to the caveolae. One possibility is that 19aa segment located at the C-termini of COX2, which is not responsible for the catalytic activity of the enzyme and whose function still remains highly unknown [2, 53], could play a role in differential trafficking of COX2 enzyme. While these are relevant concerns and would be interesting to investigate, they were beyond the scope of the present study and will be addressed in future studies.

Our previous studies, have reported that COX2 has tumorogenic effects in LNCAP human prostate cancer epithelial cells [38]. This is consistent with several studies from other groups, reporting that COX2 and its metabolites modulate mitogenic responses in human prostatic carcinoma cell lines of PC3, LNCAP and DU145 [39-43]. Therefore we conducted our studies of COX2 activity measurements in DU145 prostate cancer cells. However while all of our studies in DU145 cells are performed in the presence of FYN, here we also show that LYN another Src family member kinase, is also capable of phosphorylating COX2 enzyme but on Y120 residue located on the dimerization domain of the enzyme. Studies have shown that there could be cross-talk between monomers of COX2 homodimers; where one monomer serves as the allosteric subunit enabling the catalytic subunit to catalyze the reaction [54-56]. Thus, it is quite possible that introduction of a negative charge will alter COX2 dimerization and either diminish or increase PG production. Therefore, we can't exclude that other Src family kinases could also modulate the activity of the enzyme. Future studies could be designed, to look at additional Src family kinases in regulation of COX2 enzyme activity in prostate cancer cells. We further found that FYN regulates the activity of COX2 by phosphorylating Y446 residue; located on the catalytic domain of the enzyme. While multiple residues, including tyrosine residues situated close to the active site of COX2 have been directly involved in enzyme catalysis, Y446 has not been shown to be one of the residues involved in PG biosynthesis [2]. Therefore, it is still not clear whether effect of phosphorylation at the Y446 site on the activity of the enzyme is direct. It might be possible that phosphorylation of this site either allosterically regulates PG biosynthesis, or rather controls subcellular localization of COX2 and its association with other proteins.

Finally, many studies correlate an overexpression of COX2 with prevention of apoptosis and induction of angiogenesis in different types of cancer cells [18, 57-62]. We have previously shown that that COX2- mediated prostaglandin synthesis protects human prostate cancer cells and other cells from apoptosis [38, 63, 64]. Thus, we propose that COX2 tyrosine phosphorylation resulting in increased PG production, might be important in regulation of such processes associated with tumorogenesis.

In this study we propose a novel mechanism of modulation of the PG biosynthetic pathway: the regulation of COX2 activity by tyrosine phosphorylation by the cytoplasmic tyrosine kinase Fyn. Further, studies have shown through a combination of data mining, immunobloting, RT-PCR, and immunohistochemistry that FYN expression is upregulated in the progression to cancer from both normal epithelium and prostate intraepithelial neoplasia (PIN). This study also reported that while levels of FYN were increased in prostate cancer, other Src kinases either did not show consistent upregulation, or were elevated to a lesser degree than FYN [65]. In prostate cancer, FYN and other SFKs have been shown to mediate extracellular interactions driven by various molecules including IL-8, c-Met, EGFR and integrins, contributing to metastatic transformation of prostate cancer [66]. Additionally, studies have reported that overexpression of FYN results in promotion of the anti-apoptotic activity of Akt [67-69], and Akt activation is detected in prostate cancer [70]. However the mechanisms of how aberrant FYN function leads to dysregulated Akt activation in prostate cancer are unknown. Since increased COX2 activity and prostaglandin signaling are associated with activation of anti-apoptotic pathways, it is possible that COX2 can be an important mediator in this signaling pathway. This is consistent with other studies showing that FYN can partner with other signaling molecules such as FAK and paxilin, which are upregulated in prostate cancer overexpressing FYN [65]. Further, our data suggest a unique mechanism of COX2 regulation by FYN in prostate cancer, since FYN expression in comparison to other Src family kinases has been shown to be upregulated in the progression to cancer from both normal epithelium and PIN [65]. Additionally, while we did not study the effect of other Src kinases on COX2 activity, it is possible that LYN might also have an effect on COX2 activity, as it phosphorylates COX2 on dimerization domain and it has been established that there is a cross-talk between monomers of COX2 homodimers [54-56]. In summary, our studies suggest that FYN/COX2 interaction is a novel molecular target in prostate cancer. We further suggest that regulation of COX2 activity by FYN could contribute to the progression of prostate cancer. Since this novel mechanism of cellular regulation of COX2 activity by FYN should take place predominately in cancer cells and particularly in prostate cancer cells, which are characterized by enhanced expression of FYN in contrast to other members of the Src family [65], this will allow us to target COX2 in cancers cells without affecting COX2 function in normal cells. Overall, these studies are important because they uncover signaling mechanisms causative to cancer progression, offer novel targets for inhibition of COX2 and treatment of prostate cancer and set the base for the development of novel diagnostic tools.

## METHODS

### Plasmids and Adenovirus constructs

Human COX2 pEZ-M02 vector (GeneCopoeia) was used to make COX2 site directed mutagenesis using a QuikChange Kit (Agilent Technologies), according to the manufacturer's instructions. All mutations were confirmed by restriction enzyme digest and DNA sequencing. Adenovirus constructs encoding rat COX2 were constructed by the Medical College of Wisconsin adenoviral core. FYN, GFP and Null adenovirus constructs were purchased from Vector Biolabs.

### Cells and transfections/transductions

Prostate cancer cell lines (DU145) and SV40-transformed human mesangial cells (HMC) were cultured in RPMI 1640 growth media, supplemented with 10% fetal bovine serum (FBS) and 100 units/ml streptomycin/penicillin/glutamine (PSIG) (Invitrogen). HEK 293T cells were cultured in high glucose DMEM growth media, supplemented with 10% FBS and 100 units/ml PSIG (Invitrogen). DU145 and HEK 293T cells lines were obtained from American Type Culture Collection (ATCC) and HMC were kindly provided by Jean-Daniel Sraer (INSERM Unite 64, Hôpital Tenon, Paris, France). All cell lines were maintained at 37 °C in a humidified atmosphere of 5% CO_2_ and 95% air. Plasmid transfections were performed using lipofectamine 2000 reagent (Invitrogen). Adenovirus mediated infections were carried out at a multiplicity of infection (moi of 10) for 1h at 37 °C with periodic shaking, followed by addition of complete growth medium.

### HPLC-MS-MS identification of COX2 cross-linking adducts in HMC cells

HMC were infected with adenovirus encoding rat COX2 at m.o.i. of 200 and further incubated in FBS containing medium for 48hrs. To identify the proteins involved in regulation of COX2 activity, COX2 adducts were isolated by immunoprecipitation with COX2 antibodies and subjected to tandem mass spectrometry. COX2 adducts were immunoprecipitated with COX2 antibodies, separated using SDS-PAGE and silver stained gel slices, corresponding to positions of high molecular weight bands recognized by COX2 antibodies were digested with Glu-C prior to analyzis by nano-LC-MS/MS at Scripps Center for metabolomics and Mass Spectrometry. A search against mammalian database indicated the presence of COX2 and several other proteins including FYN.

### Cyclooxygenase activity assays

### Measurement of prostaglandins by liquid chromatography–electrospray ionization-mass spectrometry (LC–ESI-MS)

To measure PG levels in DU145 cells overexpressing FYN or GFP, cell homogenates (homogenization buffer pH 7.7, containing 250 mM Sucrose, 1 mM EDTA, 1 mM monobasic KH_2_PO_4_, 9 mM dibasic K_2_HPO_4_ and 0.1 mM PMSF) were incubated with 40 μM AA (Cayman Chemical) for 1h in a shaking water bath at 37 °C super fused with 100% O_2_, followed by addition of 1M formic acid to stop the reaction. Alternatively, PG levels were also measured by incubating intact cells with 10 μM AA in 5ml HEPEs buffer (pH 7.4, 10 mM HEPEs, 155 mM NaCl, 5 mM KCl, 1.8 mM CaCl_2_, 1 mM MgCl_2_, and 5 mM glucose) at 37 °C for 10 min, followed by addition of 10 μM A23187 calcium ionophore for another 10 min to halt the reaction. PG's were then extracted by solid-liquid phase extraction with ethyl acetate on C18 Bond Elut SPE columns, along with [^2^H_4_]PGD_2_ used as an internal standard, and further subjected to liquid chromatography–electrospray ionization-mass spectrometry (LC–ESI-MS, Agilent 1100 LC/MSD, SL model). The samples were separated on a reverse phase C_18_ column (Kromasil, 250×2 mm, Phenomenex) using water–acetonitrile with 0.1% acetic acid as a mobile phase at the flow-rate of 0.3 ml/min. The mobile phase gradient started at 35% acetonitrile, linearly increased to 100% acetonitrile in 35 min, and held for 10 min. Drying gas flow of electrospray chamber was 12 l/min, drying gas temperature was 350 °C, nebulizer pressure was 35 p.s.i.g, vaporizer temperature was 325 °C, and fragmentor voltage was 120 V. The detection was made in the positive mode. For quantitative measurement, the *m*/*z* 353, 335, 335, 319, 317, 357, 339, 339, and 323 were used for measuring of 6-keto-PGF_1α_, PGD_2_, PGE_2_, PGF_2α_, PGJ_2_, [^2^H_4_]6-keto-PGF_1α_, [^2^H_4_]PGD_2_, [^2^H_4_]PGE_2_, and [^2^H_4_]PGF_2α_, respectively. [^2^H_4_]PGD_2_ was used as an internal standard for quantitation of prostaglandins. The standard curves were typically constructed over the range of 5 to 1000 pg per injection. The concentrations of these prostanoids in the samples were calculated by comparing their ratios of peak areas of compounds to the internal standards with the standard curves. The measured prostaglandin levels were further normalized to the protein concentrations.

### Peroxidase Endpoint Assay

Recombinant COX2 (Sigma-Aldrich) (2 μg) heat inactivated at 65 °C degrees for 25 min to remove contaminating kinases, was incubated with recombinant active FYN kinase (Origene) in the presence of ATP for 1h at room temperature, to allow for phosphorylation of COX2 by FYN. Afterwards, the sample reaction was incubated with 100 μM AA, 1 μM haematin and peroxidase activity of COX2 was measured spectrophotometrically with 170 μM *N,N,N′,N′*-tetramethylphenylenediamine (TMPD)(Sigma-Aldrich), as the reducing cosubstrate over a 5 min period. Total COX2 activity was determined using the TMPD extinction coefficient (0.00826 μM^−1^), and control wells containing no COX2 were subtracted as background non enzymatic oxidation.

### Prostaglandin E2 Biotrak Enzymeimmunoassay (EIA)

DU145 or HEK 293T cells transfected with wild type or mutant COX2 or transduced with AdFYN, were stimulated with 30 μM AA for 15 min in appropriate serum free growth media at 37 ° C. Total cellular PGE_2_ was then measured in the cell lysates using PGE_2_ enzyme immunoassay kit (EIA) (GE Healthcare), following the protocols recommended by the company.

### Immunoblotting

For immunoblotting experiments, cells were lysed in Triton X-100 lysis buffer (20 mM Tris–Cl, 150 mM NaCl, 1% Triton X-100 and 10% glycerol; pH 7.5) supplemented with protease and phosphatase inhibitors (Sigma-Aldrich), and protein concentrations were determined by BCA assay (Thermo Scientific). Protein expression was analyzed by 7.5% SDS-PAGE, which was electrophoretically transferred onto PVDF membrane, blocked in 5% milk or 3% BSA (bovine serum albumin) in 1X TBST, and probed with primary antibodies indicated in the figure legends. Species specific horseradish peroxidase-conjugated secondary antibodies were used, and proteins were detected by chemiluminescence kit (Amersham). Following primary antibodies were used: α- COX2, COX1, FYN (Santa Cruz Biotechnology), α-phosphotyrosine 4G10 antibodies (Millipore). Peroxidase-conjugated secondary antibodies used included, donkey α -goat and goat α-mouse immunoglobulins (IgGs) (Bio-Rad).

### *In vitro* kinase assay

Recombinant COX2 (2 μg), heat inactivated at 65 °C degrees for 25 min, was incubated with recombinant active FYN, LYN or JAK2 kinases (Millipore) in 25 μl reactions (50 mM Tris pH 7.4, 10 mM MgCl_2_, 1 mM dithiothreitol (DTT), 5 μM ATP (Sigma- Aldrich)) for 1h at room temperature. Reactions were then terminated by addition of 6X laemli buffer, resolved by SDS –PAGE, and immunoblotted with non-selective phosphor-tyr specific antibodies.

### Identification of phosphorylation sites on COX2 by multi-stage fragmentation tandem mass spectrometry

*In vitro* kinase assays using recombinant COX2 and recombinant active FYN kinase were performed as described above. The sample was then subjected to buffer exchange with 250 mM Ammonium bicarbonate(NH_4_HCO_3_), reduced with 10 mM dithiothreitol for 30 minutes, alkylated with 55 mM iodoacetamide for 45 minutes, and processed by tryptic digestion using 1 μg Trypsin Gold, mass spectrometry grade (Promega) at 37°C overnight. The resulting peptides were then vacuum dried and desalted with ZipTip C_18_ pipette tips (Millipore) according to the manufacturer's protocol. The protein digests were analyzed using an LTQ-Orbitrap Velos mass spectrometer (Thermo Scientific) interfaced with a NanoAquity UPLC (Waters Corp.) system and an autosampler coupled to a nano-electrospray ionization source. Peptides were injected onto a 10 cm by 75 μm inner diameter column packed in-house with 5 μm Magic C_18_ beads (100Å pore size, Polymicro Technologies). The solvents A and B used for chromatographic separation of peptides were 2% acetonitrile in 0.1% formic acid and 98% acetonitrile in 0.1% formic acid, respectively. The peptides were resolved at the rate of 200 nL/min, with a gradient of: 0-2 min 2% B, 2-5 min 2-20% B, 5-120 min 20-40% B, 120-135 min 40-60% B, 135-150 min 60-70% B, 150-158 min 70-98% B. 98% B was held for 4 min, then switched to 98% A over 8 min and held for another 10 min. The ions eluted from the column were electrosprayed at a voltage of 1.75 kV. The ion transfer temperature was kept at 250°C. No auxiliary or sheath gas was used. MS1 scans were detected in the FTMS section of the Orbitrap Velos in profile mode at a resolution of 30,000 (full width of peak at half-maximum at 400 m/z). Full scans were followed by the fragmentation by collision-induced dissociation of the 10 most intense ions in the LTQ analyzer. Neutral loss triggered multistage activation for simultaneous fragmentation of neutral loss product and precursor ions was enabled at *m/*z of -98, -49, -32.7, and -24.5, corresponding to a neutral loss of the phosphate moiety from +1, +2, +3, and +4 charged ions. Dynamic exclusion was enabled with a repeat duration of 30 s and exclusion duration of 60 s. Data analysis was performed using MASCOT algorithm against the Uniprot human database. Peptides identified with MASCOT score of 50 or above were considered potential positive identifications. Finally, the peptides listed were manually verified for correct identification by comparing the experimental spectra with the theoretical *b-* and *y-* ion spectra.

### Statistical Analysis

When necessary, data were expressed as mean and standard error of mean values. To account for experiment (day) as a random effect, linear mixed model with log transformation was used to compare effects between experimental (FYN) and control (GFP/Null) groups on prostaglandin levels in both DU145 and HEK 293T cells. *In vitro* COX2 activity measurements indicated a proportional response, therefore a log-transform of COX2 enzyme activity was used and two-way ANOVA was performed to compare FYN+COX2 to COX2 control group. P<0.05 was used as the criterion for statistical significance.
